# Macrophage-Mediated Lymphangiogenesis: The Emerging Role of Macrophages as Lymphatic Endothelial Progenitors

**DOI:** 10.3390/cancers4030618

**Published:** 2012-06-27

**Authors:** Sophia Ran, Kyle E. Montgomery

**Affiliations:** Department of Medical Microbiology, Immunology and Cell Biology, Southern Illinois University School of Medicine, 801 N. Rutledge, Springfield, IL 62794, USA

**Keywords:** lymphatic endothelial cell progenitors, tumor associated macrophages, lymphangiogenesis, lymphvasculogenesis, lymphatic metastasis

## Abstract

It is widely accepted that macrophages and other inflammatory cells support tumor progression and metastasis. During early stages of neoplastic development, tumor-infiltrating macrophages (TAMs) mount an immune response against transformed cells. Frequently, however, cancer cells escape the immune surveillance, an event that is accompanied by macrophage transition from an anti-tumor to a pro-tumorigenic type. The latter is characterized by high expression of factors that activate endothelial cells, suppress immune response, degrade extracellular matrix, and promote tumor growth. Cumulatively, these products of TAMs promote tumor expansion and growth of both blood and lymphatic vessels that facilitate metastatic spread. Breast cancers and other epithelial malignancies induce the formation of new lymphatic vessels (*i.e*., lymphangiogenesis) that leads to lymphatic and subsequently, to distant metastasis. Both experimental and clinical studies have shown that TAMs significantly promote tumor lymphangiogenesis through paracrine and cell autonomous modes. The paracrine effect consists of the expression of a variety of pro-lymphangiogenic factors that activate the preexisting lymphatic vessels. The evidence for cell-autonomous contribution is based on the observed tumor mobilization of macrophage-derived lymphatic endothelial cell progenitors (M-LECP) that integrate into lymphatic vessels prior to sprouting. This review will summarize the current knowledge of macrophage-dependent growth of new lymphatic vessels with specific emphasis on an emerging role of macrophages as lymphatic endothelial cell progenitors (M-LECP).

## Abbreviations

TAM(s): tumor-associated macrophage(s)
M-LECP: macrophage-derived lymphatic endothelial cell progenitor(s)
LN(s): lymph node(s)
BM: bone marrow
LEC(s): lymphatic endothelial cell(s)
IFP: interstitial fluid pressure
Prox1: prospero-related homeobox-1
VEGFR: vascular endothelial growth factor receptor
LECP: lymphatic endothelial cell progenitor(s)
LVD: lymphatic vessel density
VEGF: vascular endothelial growth factor
NF-κB: nuclear factor-kappaB
BEC: blood vascular endothelial cell(s)
IBC: inflammatory breast cancer
MMP: matrix metalloproteinase(s)
CDL: clodronate liposomes
GFP: fluorescent protein

## 1. Introduction

Lymphangiogenesis is the process of formation of new lymphatic vessels. The studies in the past decade brought ample new knowledge on the formation of new lymphatic vessels during both embryonic development and in adulthood. This information is important because the proper function of the lymphatic system is crucial for maintaining homeostasis, normal metabolism, and immune responses. Consequently, disorders of the lymphatic system that include both lymphatic functional or structural deficiency as well as pathological outgrowth affect millions of patients worldwide. Currently, most of the disorders of the lymphatic system have limited or no therapeutic solutions. Clinical management of these diseases is achievable only if the mechanisms of the postnatal lymphangiogenesis are well understood. Although the details of these mechanisms only recently began to emerge, a plethora of prior observations clearly show that postnatal lymphangiogenesis is primarily associated with chronic inflammation, a process that on the cellular level is chiefly regulated by macrophages. In addition to chronic inflammatory disease, macrophages also play a prominent role in tumor progression and metastasis. Tumor metastasis was previously attributed to the ability of tumor-associated macrophages (TAMs) to promote angiogenesis (*i.e*., the formation of blood vessels) that facilitates hematogenous spread. However, most epithelial malignancies metastasize first to the regional lymph nodes (LNs) through lymphatic vessels prior to dissemination through blood vasculature. These observations suggest that tumor macrophages play a critical role in promoting not only angiogenesis but also lymphangiogenesis that leads to lymphatic metastasis.

In this review, keeping in consideration a potentially broad readership, we first present basic information about the structure of lymphatic vessels, markers expressed on lymphatic endothelial cells (LECs), embryonic development of lymphatic vessels, and growth of new lymphatics in adult. Additionally we introduce the molecular mechanisms underlying inflammatory/tumor lymphangiogenesis and the significance of new lymphatics in tumor spread. This introduction will sets-up the stage for the main focus of this review on differentiation of myeloid cells to lymphatic endothelial cell progenitors (M-LECP) and their roles in adult lymphangiogenesis.

### 1.1. Structure, Function, and Organization of the Lymphatic Vasculature

The lymphatic vascular system is a hierarchical network of vessels comprised of blind-ended capillary beds and larger collecting vessels that form a unidirectional system draining most soft tissues of the body. Lymphatic vessels are found in all vascularized tissues in vertebrates except the bone marrow (BM) and brain as larger body size necessitates a secondary vascular system to maintain fluid homeostasis [1]. The initial lymphatic capillaries are specifically designed to absorb fluid; accordingly, they are made up of a single layer of overlapping lymphatic endothelial cells (LEC) [2] with button-like intercellular junctions [3], discontinuous basement membrane, and specialized filaments that anchor these vessels to the extracellular matrix [4]. Lymphatic uptake of interstitial fluid, a critical function to maintain the balance of interstitial fluid pressure (IFP), is facilitated primarily by these anchoring filaments and functional “gap sites” in the LEC monolayer [5]. Lymph entering the initial lymphatic capillaries is propelled forward into the larger collecting lymphatics by cyclical tissue deformation such as body movement, breathing, and skeletal muscle contraction [6]. Lymph propulsion through higher order lymphatic collectors and trunks is facilitated by unidirectional valves and contractions of smooth muscle cells [1]. After being filtered through a series of LNs, most proteins, lipids, and macromolecules making up the lymph are ultimately returned to venous circulation through the thoracic duct that empties its contents into the left subclavian vein.

In addition to maintaining IFP, the lymphatic vasculature regulates lipid adsorption in the gut [7], facilitates transports of hormones and cytokines produced in adipose tissue [8], and performs many immune functions including antigen presentation [9] and immune cell trafficking [10]. The lymphatic vessels serve as the primary pathway to transport tissue-absorbed soluble antigens, antigen-presenting dendritic cells, and lymphocytes into regional LNs, which is the first step for mounting an adaptive immune response [1]. During either sterile (*i.e*., due to injury or tumor) or pathogen-induced inflammation, the necessity to maintain fluid, protein, and lipid balance, as well as to interact with the immune system, is drastically increased. This necessity is likely the primary driving force for the formation of new lymphatics because the needs of an inflammatory site, particularly, under prolonged conditions, may well exceed the functional capacity of the local preexisting vessels. Thus, generation of new lymphatic vessels is an appropriate tissue response to growing fluid imbalance and leukocyte trafficking demands imposed by chronically inflamed sites and tumors.

### 1.2. Markers of Lymphatic Endothelial Cells (LECs)

In recent years, research of the lymphatic vasculature has been greatly facilitated by the discovery of several proteins expressed primarily on LECs. The six most frequently used markers that discern between blood and lymphatic vessels are: vascular endothelial growth factor receptor-3 (VEGFR-3), Prospero-related homeobox-1 (Prox1), a hyaluronan receptor LYVE-1, a mucin-type transmembrane glycoprotein podoplanin, integrin alpha-9, and neuropilin-2 (NRP2).The earliest to be described was VEGFR-3 [11], a tyrosine kinase receptor that is the key protein that regulates lymphangiogenesis in adult [12]. Throughout adulthood, VEGFR-3 is mainly found in LECs except occasional expression in inflamed [13] and tumor blood vessels [14], circulating LEC progenitors [15,16], activated macrophages [17,18,19,20,21,22,23,24], and some malignant cells [25]. LYVE-1 [26] is one of the most specific markers of the adult LEC with limited expression on sinusoidal blood vessels in the liver [27] and spleen [26]. LYVE-1 is absent in resting macrophages, and its expression in activated ones [19,23,28,29,30] might signify macrophage transdifferentiation to LECP [30]. An additional marker that is expressed predominately in LECs is the transcription factor Prox1. This marker, however, is also expressed in non-endothelial cells such as hepatocytes and neurons [31]. Another specific marker is podoplanin (also known as T1α/podoplanin and D2-40), a protein mainly found on the LEC surface with minor expression in a few other cell types [32]. LEC can also be identified by integrin alpha-9 that is expressed on lymphatic but not blood vascular endothelial cells [33]. However, besides vasculature, integrin alpha-9 has a relatively broad expression pattern in mesenchymal, epithelial and other cell types [33,34,35]. Lastly, neuropilin-2 (NRP2), a neuronal semaphorin receptor [36], is expressed predominantly on LECs with minor expression on veins [37]. NRP2 physically interacts with VEGFR-3 and serves as a co-receptor for VEGF-C/-D [38]. Additionally, NRP2 is upregulated in the tip cells of sprouting lymphatics and is required for sprout initiation during postnatal development [39]. Although none of these markers has an exclusive specificity to LEC, when used in combination, they unequivocally distinguish lymphatic vessels from blood vasculature.

### 1.3. Development of the Lymphatic Vascular Network During Embryogenesis

The purpose of this review is to discuss the role of macrophages in adult lymphangiogenesis. To better understand this process, the authors would like first to highlight the main points of the embryonic lymphatic development without going through detailed information about this process available in recent reviews published elsewhere [40,41,42]. Here, we will mainly focus on two aspects of embryonic lymphatic development as they might be recapitulated during adult lymphangiogenesis: (1) transcriptional control of venous-derived LECs; and (2) the potential of non-venous or myeloid cells to differentiate into LECs.

The prevalent view is that LECs originate from endothelial cells of the cardinal vein [43] and that this process begins around the day E8.5 with the expression of transcription factor CouptfII [44]. This is followed by expression of Sox18 at the day E9.0, a transcription factor that signifies the onset of lymphatic differentiation [45]. Sox18 then drives the expression of transcription factor Prox1 that is considered the “lymphatic master switch” indicating its central role in commitment to the lymphatic lineage [46]. Subsequently, the pre-committed ECs continue to gain autonomy through a stepwise process that includes upregulation of lymphatic genes, downregulation of venous markers, and budding from the cardinal vein to form the first lymphatic structures called lymph sacs [41]. Lymph sac-derived LECs migrate and form nascent vessels that after remodeling serve the foundation for the postnatal lymphatic network [43].

Although this model of embryonic lymphangiogenesis is largely supported by multiple studies, some evidence indicates that the peripheral lymphatic vessels and superficial parts of lymph sacs might be of non-venous origin. This was shown in several species demonstrating that embryonic LECs can derive from mesenchymal cells called lymphangioblasts that are of non-venous [47,48,49,50,51] or myeloid origin [52]. For instance, Prox1 and VEGFR-3 positive lymphangioblasts were detected in mesodermal tissue in avian embryos [49,50] where these cells contributed to newly-formed lymphatic vessels in the wing [49,50] and participated in the formation of jugular lymph sacs along with venous-derived LECs [50]. Similarly, murine embryonic mesenchymal cells positive for Prox1, LYVE-1, and macrophage marker F4/80 were detected within the lymph sacs and developing peripheral lymphatic vessels [52]. It is, therefore, conceivable that genetic programs in myeloid or other non-venous LECP might be potentially reactivated in precursors that contribute to lymphangiogenesis in adult.

## 2. Generation of New Lymphatic Vessels in the Adult

The current dogma is that the formation of new adult lymphatic vessels can occur either through *lymphangiogenesis* (*i.e*., sprouting from preexisting vessels), or through *lymphvasculogenesis*, (*i.e*., *de novo* formation of lymphatic vasculature from LECP that originate from BM [15,16,53] or another non-vascular source [20]). Studies on embryonic development of the lymphatic system suggest that both processes are not necessarily mutually exclusive by demonstrating that at least some segments of the lymphatic system might have a dual venous and mesenchymal base. A similar combination of the two basic processes of vessel formation may also occur in adults, although without formal investigation, clear evidence for this idea is still missing. Below, we review the evidence that inflammation and tumors induce new lymphatic vessel formation and that adult BM-derived myeloid progenitors promote lymphangiogenesis, lymphvasculogenesis or both processes.

### 2.1. Inflammation-Induced Lymphangiogenesis

Chronic inflammatory conditions are typically associated with increased lymphatic vessel density (LVD) (Table 1). This is illustrated by findings in both clinical studies and experimental models of inflammation including psoriasis [54], inflammatory bowel disease [55,56], rheumatoid arthritis [57], atherosclerosis [58], skin irradiation [59], and cancer [60].

For instance, inflammatory lymphangiogenesis shown by increased LVD was detected in patients rejecting renal transplants [61]. Lymphangiogenesis in this situation was attributed to VEGF-C derived primarily from macrophages [20]. This notion is supported by a study in breast cancer patients receiving radiotherapy that showed a significant correlation between the density of VEGF-C^+^ macrophages and LVD [59]. Inflammation-induced lymphangiogenesis has also been shown in many experimental models including those of wound healing [12], corneal injury [62], skin inflammation [63], peritonitis [64], and chronic inflammation in airways [65]. These models reproducibly showed extensive lymphangiogenesis triggered by macrophage-derived inflammatory mediators suggesting that VEGF-C and other products of activated macrophages are major contributors to the postnatal formation of new lymphatics. Additionally, several models have shown inflammation induced qualitative changes in the lymphatic network such as enlarged, dysfunctional vessels [64], and remodeling of VE-cadherin junctions between LECs [66] (Table 1).

**Table 1:** Examples of inflammation induced quantitative and qualitative changes in LVs.

Model/Condition	Quantitative measure	Qualitative change	Ref.
Psoriasis (H)	2–6 fold increased LVD and ~2 fold increased Ki-67 index	N/A	[54]
Inflammatory bowel disease (H)	~2–3 fold increase in LVD	N/A	[56]
Irradiated skin (H)	18% increase in total LVD and 44% increase in vessels <10 µm in diameter	N/A	[59]
Kidney transplant rejection (H)	>50 fold increased LVD in grafts undergoing rejection	N/A	[61]
Breast cancer (H)	LVD was 12 fold higher in tumors compared to benign lesions	N/A	[67]
UVB irradiation of skin (m)	2–3 fold increased LV area and size but no increase in LV number	Hyperplastic vessels associated with increased macrophage infiltration	[63]
LPS induced peritonitis (m)	~2–4 fold increased LVD in diaphragm; 2.4 fold more proliferating LECs; 17 fold more vessel sprouts	LVs were enlarged and LV network patterning was atypical; increase in randomly oriented branching; new LVs were dysfunctional	[64]
TG stimulated peritonitis (m)	1.9 fold increased LVD in diaphragm	N/A	[68]
Chronic airway inflammation (m)	LVD and LV sprouts increased many folds (roughly 10 fold) in trachea	N/A	[65]
Chronic airway inflammation (m)	LVD increased >10 fold in trachea	VE-cadherin LEC junctions are remodeled, intermittent buttons give way to continuous zippers	[66]

LV, lymphatic vessels; (H), human; LVD, lymphatic vessel density; (m), mouse; LPS, lipopolysaccharide; TG, thioglycolate.

### 2.2. Molecular Mediators of Inflammatory Lymphangiogenesis

Given the fact that inflammation is the primary trigger of pathological lymphangiogenesis, it is not surprising that most known pro-lymphangiogenic factors are either inflammatory cytokines or downstream products of inflammatory pathways activated by transcription factors of the nuclear factor-kappaB (NF-κB) family [69]. The main NF-κB complexes that transmit inflammatory signals are p50/p65 heterodimers or homodimers of these proteins [70]. Some NF-κB-transcribed genes stimulate lymphangiogenesis directly (e.g., vascular endothelial growth factor A (VEGF-A) [71] and VEGF-C [72]) while others (e.g., IL-1β [73], TNF-α [73], and COX-2 [74]) act indirectly by upregulating lymphangiogenic factors. NF-κB proteins are also known to activate the promoter of VEGFR-3 [68], the key inducer of lymphangiogenesis. The central role of VEGFR-3 in generation of new lymphatic vessels was shown by significantly reduced LVD after blockade of VEGFR-3 at inflammatory [65], wound healing [12], and tumor sites [75].

VEGFR-3 can also be regulated by Prox1, a transcription factor that specifies the fate of LEC during embryogenesis [46]. Prox1 appears to perform a similar function in adult endothelial cells as evidenced by up- and downregulation of VEGFR-3 following forced Prox1 overexpression or silencing in blood vascular endothelial cells (BEC) [33] and in LEC [76], respectively. Also, NF-κB synergizes with Prox1 in regulation of VEGFR-3 expression [68], which shows an additional pro-lymphangiogenic mechanism induced by inflammation.

Inflammation-mediated increase in VEGFR-3 is probably needed to increase the responsiveness of preexisting lymphatic vessels to VEGF-C and VEGF-D. This supposition is based on the fact that while these lymphangiogenic factors are present at very high concentrations at the inflammatory site being produced by a variety of recruited and local cells [65,73,77], the level of surface expression of VEGFR-3 in LEC eventually determines the response rate. The high level of VEGFR-3 on LEC surface collectively regulated by Prox1 and NF-κB is therefore crucial for mounting robust lymphangiogenesis in response to inflammatory stimuli.

Another event that enhances inflammatory lymphangiogenesis is generation of mature forms of VEGF-C/-D through proteolytic processing mediated by plasmin or furin present at high concentrations at inflammatory sites [78,79]. Mature VEGF-C/-D have increased affinity to VEGFR-3 and novel ability to bind VEGFR-2 [80] expressed on both LEC and BEC [81]. Binding of mature VEGF-C/-D to VEGFR-2 and VEGFR-3 expressed in LEC leads to formation of heterodimer of respective receptors, an event that was reported to enhance signal transduction and activation of LEC [81,82].

Another important promoter of lymphangiogenesis is VEGF-A, initially thought to be an exclusively angiogenic factor [83,84]. NF-κB potently upregulates VEGF-A [71] whose elevated expression is noted in a variety of chronic inflammatory conditions such as psoriasis [85], rheumatoid arthritis [86], inflammatory bowel disease [87], chronic airway inflammation [88], and cancer [89]. VEGF-A dependent inflammatory lymphangiogenesis was first demonstrated in mice treated with adenovirus encoding this factor [90], and subsequently shown in models of corneal injury [91,92] and skin cancer [93]. Tumor lymphangiogenesis induced by VEGF-A has been shown in a mouse model of T241 fibrosarcoma [94], as well as in MDA-MB-231 and MDA-MB-435 models of breast cancer [95]. Neutralizing VEGF-A substantially reduced LVD and metastasis in the MDA-MB-231 breast tumor model [95]. Evidence from other tumor models showed that VEGF-A can induce both intratumoral [93] and peritumoral [94] lymphatic vessels that, in turn, facilitate lymphatic metastasis. The pro-lymphangiogenic effect of VEGF-A can be mediated in a direct manner through binding to VEGFR-2 as evidenced by proliferation and migration of VEGFR-2-positive LEC *in vitro* [96]. However, VEGF-A also recruits macrophages [97] that produce high levels of the pro-lymphangiogenic factors VEGF-C/-D [91], thus acting as an indirect enhancer of lymphangiogenesis. Additionally, VEGF-A was shown to increase VEGFR-3 expression in LEC [98] whereas an anti-VEGF-A antibody was shown to inhibit VEGFR-3 expression [95]. These studies suggest that VEGF-A contribution to regulation of VEGFR-3 expression might be an additional mechanism to promote formation of lymphatics in VEGF-rich environment.

Angiopoietin-2 (Ang-2) is another inflammatory mediator with pro-lymphangiogenic activity. Ang-2 and related protein, Ang-1, are ligands for the tyrosine kinase receptor, Tie2 [99], expressed in both BEC and LEC [100]. Although Ang-1 and Ang-2 have antagonistic functions in BEC activation [101], they both play positive roles in induction of lymphangiogenesis [102] as illustrated by the requirement for Ang-2/Tie2 signaling for embryonic lymphatic development [103,104]. Analogous to the complex pro-lymphangiogenic effect of VEGF-A, Ang-2 promotes lymphatic growth by several mechanisms that include direct activation of Tie2, indirect increase in VEGFR-3 expression [95], and enhancement of LEC activities via crosstalk between Tie2 and VEGFR-2 pathways [105]. Ang-2 also activates Tie2-positive macrophages (TEMs), a highly pro-angiogenic subset of circulating myeloid cells that infiltrate tumors and overexpress VEGF-A and MMP-9 [106]. These proteins promote both angio- and lymphangiogenesis suggesting that Ang-2-activated TEMs might contribute to both processes.

Several inflammatory cytokines can promote lymphangiogenesis either directly or indirectly, by upregulating VEGF-C/-D. For instance, IL-7 increases expression of VEGF-D and induces transcription of lymphatic-specific genes such as Prox1, LYVE-1, podoplanin and VEGFR-3 [107]. IL-7 was shown to increase proliferation, migration, and tube formation of endothelial cells *in vitro* [107] and the growth of LYVE-1^+^ vessels in tumor-containing matrigel plugs *in vivo* [108]. IL-7 correlates with lymphatic metastasis in breast cancer patients [109] suggesting that this results from IL-7 induced tumor lymphangiogenesis. Another interleukin, IL-3, has also been shown not only to induce LEC proliferation and migration but also to enhance similar effects of VEGF-C [68]. A possible synergistic mechanism of VEGF-C and IL-3 might be mediated through its ability to activate the NF-κB pathway which, in turn, promotes transcription of VEGFR-3 [68].

Other factors that increase inflammatory lymphangiogenesis include fibroblast growth factor (FGF)-2 [62], platelet-derived growth factor (PDGF) [110], insulin-like growth factor-1 and -2 (IGF-1,-2) [111], hepatocyte growth factor (HGF) [112], growth hormone [113], fasting-induced adipose factor (FIAF) [114], and sphingosine-1 phosphate (S1P) [115]. PDGF [110], IGF-1,-2 [111], and growth hormone [113] induce lymphangiogenesis directly, independently of VEGR-3 signaling. In comparison, factors such as FGF-2 [62] and Cox-2 [116] elicit their effects indirectly by upregulating VEGF-C. Currently, however, the pro-lymphangiogenic mechanisms cumulatively regulated by these factors and their cooperation with VEGFR-3 are incompletely understood. Additional studies are needed to better understand cross-talk between VEGFR-3-induced and other signaling pathways activated in the inflammatory environment.

### 2.3. Tumor-Induced Lymphangiogenesis

#### 2.3.1. Induction of Lymphangiogenesis in Tumors

One of the recently emerging concepts is that cancer and inflammation are inherently linked and mutually propagate each other [117]. Multiple lines of evidence demonstrate that chronic inflammation predisposes to tumorigenesis whereas established tumors invariably create a chronically inflamed site [118]. The latter is driven by three independent but interrelated processes: (1) overexpression of inflammatory cytokines and chemokines frequently observed in epithelial malignancies [119]; (2) tumor pathology (e.g., hypoxia [120], necrosis [121] and fibrosis [122]) activates chemokine production in the tumor infiltrating host cells [120,123,124]; and (3) cytokine overexpression by tumor-mobilized and activated, predominantly macrophage, immune infiltrates [125]. Cumulatively, factors derived from neoplastic, stromal, and tumor-recruited immune cells propagate a continuum of inflammatory state [126]. As stated above, many inflammatory mediators have potent pro-lymphangiogenic properties. It is, therefore, not surprising that virtually all epithelial tumors induce either intratumoral or peritumoral lymphangiogenesis and utilize newly-created lymphatic channels for metastatic spread. Studies in human breast cancers provide one of the best illustrations of the strong association between tumor-associated inflammation and formation of new lymphatics. Breast tumors show significant upregulation of VEGF-C or VEGF-D, increased division of tumor-associated LEC, and higher tumor-associated LVD than in normal mammary tissue [60].

#### 2.3.2. Correlation between Tumor-Induced Lymphangiogenesis and Metastasis

Clinical studies in breast [60] and other types of solid tumors [127] overwhelmingly show that tumor-induced lymphangiogenesis correlates with both LN and distant metastasis. For patients with early stage I and II breast tumors, LN status is the most important prognostic factor for survival. LN status is independent of tumor size, histological grade, and other clinicopathological parameters [128,129]. Studies involving 2,600 [130] and 4,350 [131] patients showed strong correlations (*p* < 0.0001) between lymphatic vessel invasion and LN metastasis. As shown by numerous studies, lymphatic metastasis strongly correlates with distant metastasis and patient survival [132,133]. Because the mortality from cancer is primarily caused by distant, not LN metastases, such findings can be interpreted as evidence for the ability of LN metastatic cells to spread systemically or, as suggested initially, as a general sign for tumor aggressiveness. However, the ample data from experimental models strongly suggest causal relationship among tumor-induced lymphangiogenesis, LN metastasis, and spread to distant organs.

The first evidence for a causal relationship between tumor lymphangiogenesis and metastasis came from the study in which VEGF-C was overexpressed in tumors with low or no innate expression. Ectopic expression of VEGF-C in both MDA-MB-435 [134] and MCF-7 [135] breast cancer models showed significantly increased intratumoral lymphangiogenesis that not only increased the incidence of LN metastasis but also the burden of lung metastases. Similar results were obtained in other models of solid tumors including lung [136], prostate [137], melanoma [17,138], gastric carcinoma [139], fibrosarcoma [138], and colorectal cancer [140]. Blockade of VEGFR-3 signaling decreased LN [136,137,141] and distant metastasis [75,137], favoring the notion that LN metastasis is an intermediate step leading to systemic dissemination. Consistent with this idea, tumor lines with naturally high VEGF-C expression preferentially undergo lymphogenous metastasis as shown in models of breast [95,142], prostate [143], and gastric carcinomas [141]. Furthermore, depletion of VEGF-C by stable shRNA in mouse breast carcinoma models drastically reduced intratumoral lymphangiogenesis as well as LN and pulmonary lesions [142,144]. Studies with experimental manipulation of VEGF-D expression showed similar results in hepatocellular [145] and pancreatic [146,147] cancer models. VEGF-A was also reported to significantly contribute to LN metastasis [93,95], and neutralizing VEGF-A significantly reduces tumor LVD [95] and metastasis to both LN and pulmonary sites [95]. Collectively, these findings indicate that tumor lymphangiogenesis is a primary cause for LN metastases that, in turn, promote systemic spread leading to shortened patient survival.

## 3. Role of Macrophages in Postnatal Formation of New Lymphatic Vessels

Macrophages are the main type of immune cells that initiate, orchestrate, and propagate chronically inflamed sites. In the context of cancer, macrophages are well-known promoters of angiogenesis [120] and hematogenous metastasis [148]. Specifically in breast cancer, TAMs are reported to secrete a variety of pro-angiogenic proteins [149] and metalloproteinases that facilitate migration and invasion of both neoplastic cells [150,151] and BECs [150,152]. While the effects of the TAM products on hematogenous metastasis are well-established, the evidence for their roles in promoting lymphatic metastasis has begun to emerge only recently. Myeloid cells contribute to angiogenesis also by transdifferentiation into BEC progenitors [153]. A similar process generating macrophage-derived lymphatic progenitors has been recently shown in several models of inflammation and cancer. Thus, the current evidence suggests that pro-lymphangiogenic activities of TAMs might involve both paracrine and cell-autonomous effects, as detailed below.

### 3.1. Subtypes of TAMs Displaying Pro- or Anti-Tumorigenic Behavior

Macrophages are multifunctional immune cells that respond to a wide array of stimuli including, microbial products, inflammatory cytokines, chemokines, and growth factors (for reviews on these topics see [125,126,154,155,156]. Macrophages are strongly recruited to cancers in response to necrosis [149], hypoxia [120], and tumor-secreted chemoattractants [126,155]. They are highly plastic and can differentiate into multiple subtypes depending on signals present in local environment [156]. Activated macrophages are generally categorized as being either M1- or M2-polarized based on phenotypical characteristics and their involvement in type 1 or type 2 inflammation, respectively. Characteristics that discern between M1- and M2-polarized macrophages include effector functions, cytokine production, and expression of chemokine receptors [156]. It should also be noted that sub-division to M1 and M2 classes oversimplifies the conditions in different tumors that may induce sub-categories with M1/M2 mixed or currently unclassified features.

The classically activated M1 phenotype is stimulated by signals associated with microbial infections such an IFN-γ, TNFα, GM-CSF, and the bacterial product, LPS [125]. M1 macrophages are integral to the anti-tumor type 1 inflammatory response as they have a high capacity to present antigens, and produce pro-inflammatory cytokines that activate Th1 lymphocytes [125,126]. Additionally, M1 macrophages release high levels of toxic intermediates (e.g., nitric oxide, reactive oxygen species, TNFα) responsible for killing intracellular parasites and tumor cells [125,156]. Thus, M1-type macrophages are generally considered to be tumoricidal, although some of their factors have pro-angiogenic properties (e.g., TNFα).

Alternatively activated macrophages, referred to as M2-polarized, include several subtypes that generally suppress type 1 inflammation and promote tumor progression [125]. They activate Th2 lymphocytes, and promote wound healing, tissue remodeling, and angiogenesis. TAMs typically, but not always, undergo M2 “like” polarization and thus exhibit many pro-tumorigenic characteristics [125]. TAMs suppress adaptive immunity by secreting factors that suppress Th1 responses along with chemokines that recruit non-cytotoxic T cell subsets [125,126]. Additionally, TAMs release factors that promote tumor cell growth, survival, and migration [125]. Furthermore, TAMs promote angiogenesis and metastasis through production of growth factors such as EGF, βFGF, and PDGF; angiogenic cytokines such as VEGF-A, VEGF-C, and CXCL8; and matrix degrading enzymes such as MMP-2, MMP-7, MMP-9, MMP-12, plasmin, and urokinase plasminogen activator [125,126,157]. Many of the molecules secreted by TAMs are also lymphangiogenic (Table 2) and are discussed in detail later in this review.

**Table 2:** Role of TAM products in tumor lymphangiogenesis and lymphatic metastasis.

Name	Produced by TAMs	Evidence for lymphangiogenic activity	Ref.	Correlates with LN metastasis?	Ref.
VEGF-A	[158]	Activates LEC and directly induces lymphangiogenesis in various inflammation and tumor models	[91,159]	yes	[60]
VEGF-C	[22]	A ligand for VEGFR-3, a key inducer of lymphangiogenesis	[4,160]	yes	[60]
VEGF-D	[22]	A ligand for VEGFR-3, a key inducer of lymphangiogenesis	[4,161]	yes	[60,147]
PDGF	[125]	Direct lymphangiogenic factor in mouse cornea and PDGF-overexpressing T241 tumors	[110]	yes	[162]
Adrenomedullin	[163]	Direct lymphangiogenic factor acting through the calcitonin receptor-like receptor	[164]	yes	[165]
HGF/SF	[149]	Direct lymphangiogenic factor; the corresponding receptor c-Met is upregulated on LEC during inflammation	[166]	yes	[167]
COX-2	[74]	Induces lymphangiogenesis indirectly through PGE2 that upregulates VEGF-C	[74,116]	yes	[168]
βFGF (FGF-2)	[169]	Induces lymphangiogenesis indirectly through upregulation of VEGF-C and VEGF-D	[62,170]	yes	[171]
TNF-α	[172]	Potentially regulates lymphangiogenesis by increasing VEGF-C transcription in fibroblasts	[73]	yes	[173]
MMP-2 & MMP-9	[174]	Suppression of MMP-2-/9 inhibits LEC invasion through matrigel	[175]	yes	[176]
Heparanase	[177]	Indirectly lymphangiogenic by increasing VEGF-C expression in cancer cells	[178]	yes	[179]
Urokinase plasminogen activator system	[180]	Plasmin is indirectly lymphangiogenic by increasing maturation of VEGF-C/-D propeptides	[181]	yes	[182]
Angiopoietin-2 * (Ang-2)	[183]	Directly lymphangiogenic; activates LEC via Tie-2 receptor; overexpression of Ang-2 induces lymphangiogenesis *in vivo*	[184,185]	yes	[186]

* Ang-2 is expressed by activated macrophages, but to the best of our knowledge, Ang-2 has not been observed in TAMs specifically.

### 3.2. Association of Tumor Lymphangiogenesis and Lymphatic Metastasis with Macrophage Infiltrates

Chronic inflammation is a hallmark of breast cancer [187,188] and has been repeatedly linked to increased tumorigenesis [189,190], angiogenesis [191,192], lymphangiogenesis [65,193] and metastatic progression [194,195,196]. Clinical studies in breast, prostate, cervix, and bladder cancers showed that macrophages, master regulators of inflammation, are massively recruited to tumors and correlate with poor patient outcome [126]. Until recently, strong correlation between macrophage infiltrates and metastasis has been primarily explained by TAM-mediated release of pro-angiogenic factors that heighten angiogenesis and increase hematogenous metastasis [125]. However, many of the same proteins can also contribute to lymphangiogenesis, invasion of lymphatic vessels, and lymphogenous metastasis (Table 2). The notion that macrophages promote lymphangiogenesis is supported by clinical studies on cancers of the cervix [22], pancreas [197], lungs [198,199], breast [200], esophagus [201], and melanoma [202]. These studies have shown statistically significant associations between TAM density and tumor LVD, lymphatic invasion, and LN metastasis.

One of the first clinical studies that showed direct correlation between tumor LVD and the density of VEGF-C/-D producing TAMs was performed using specimens of cervical cancer [22]. This study found that tumor LVD correlated with VEGF-C/-D producing TAMs, and that both TAM and LVD densities correlated with LN metastasis [22]. Interestingly, VEGF-C/-D positive monocytes comprised only a fraction (~25%) of total TAMs, co-expressed VEGFR-3 and formed small clusters around lymphatic vessels [22]. The lymphangiogenic role of TAMs has been also shown in studies with pancreatic [197] and lung cancer [198]. These studies considered that TAM lymphangiogenic potential can be affected by their M1/M2 polarization. The pancreatic cancer studies used CD163/CD204 markers to distinguish M2-polarized macrophages from the entire population of CD68^+^ TAMs [197]. M2-polarized CD163/204-positive TAMs were significantly associated with increased LVD (*p* = 0.018) and decreased overall patient survival (*p* = 0.018) whereas CD68^+^ TAMs were mainly associated with LN metastasis (*p* = 0.029). In the study of lung adenocarcinoma, M1-polarized TAMs were distinguished from M2 using double staining for CD68 and iNOS [198]. Overall, 79% of TAMs were M2-polarized and significantly correlated with both peritumoral LVD (*p* = 0.009) and LN metastasis (*p* = 0.003) whereas M1-polarized TAMs were not associated with either parameter. However, high intratumoral TAM density, regardless of their subtypes, was associated with a decrease in five-year survival. An independent study of lung adenocarcinoma also showed that TAM infiltration significantly correlated with peritumoral LVD (*r* = 0.069, *p* < 0.001) and was associated with LN metastasis (*p* = 0.037) and reduced patient survival (*p* = 0.005) [199]. Interestingly, peritumoral but not intratumoral LVD correlated with TAM infiltrates [199] suggesting that macrophages primarily contribute to the lymphatic formation at the tumor periphery. Clinical associations of TAMs with lymphatic invasion [201] and LN metastasis [200,201] were also shown in esophageal [201] and breast cancers [200], although correlation with LVD in these studies was not determined.

Not all studies have found associations between TAMs and LN metastasis [203,204] or LVD [205,206]. For instance, unlike his previous study with cervical cancer, Shoppmann *et al.* found that in breast cancer neither VEGF-C producing TAMs nor VEGF-C producing tumor cells were associated with LVD [205]. Some discrepancies might relate to heterogeneity of analyzed patient cohorts. It is tempting to speculate that the anatomical location of the tumor in relation to initial or collecting lymphatics, which exhibit different responses to micro-environmental stimuli [207], would influence the degree of tumor lymphangiogenesis. Additionally, discrepancies may relate to the lack of consideration for macrophage subtypes with differential capacity to influence lymphatic formation. For instance, some studies that failed to show association between TAMs and LVD/LN metastasis did not account for M2 polarization that might determine TAM contribution to lymphangiogenesis [197,198]. However, unlike the studies described above, an additional study that distinguished M2-polarized macrophages with the marker CLEVER-1/Stablin-1 found no association between M2 macrophages and podoplanin^+^ LVD [208]. The variable results from studies that focused on M2-polarized macrophages could be due to macrophage plasticity. Depending on the combination of micro-environmental signals, M2 macrophages can polarize differentially into three subtypes (M2a, M2b and M2c) that have distinct immunological functions and molecular profiles [156]. The specific role of these macrophage subtypes in tumor lymphangiogenesis is currently unknown. The other variable that may contribute to discrepancies among experimental studies is the kinetic of the expression of M2 markers that fluctuates upon macrophage activation or interactions with other immune cells [209]. It is therefore, plausible that some discrepancies in the results of these studies might be due to functional dissimilarities in the analyzed TAM sub-groups.

### 3.3. Experimental Evidence Demonstrating Correlation Between TAMs, Increased LVD and Lymphatic Metastasis

The lymphangiogenic characteristics of TAMs have been demonstrated in many tumor models in which blocking macrophage recruitment or depleting macrophages correlated with decreased LVD and suppressed LN metastasis. For instance, blocking macrophage recruitment to orthotopic pancreatic tumors by anti-PlGF antibody reduced F4/80^+^ TAMs by 74% [210]. Importantly, this treatment resulted in a 75% decrease in LVD (*p* < 0.005), and a corresponding ~60% decrease in LN metastasis (*p* < 0.05). Tumor VEGF-C levels and blood vessel density were also decreased following this treatment [210]. Similarly, blockade of M-CSF signaling markedly reduced the recruitment of LYVE-1^+^ TAMs to osteosarcoma causing an 8–10 fold reduction in the density of peritumoral lymphatics, and ~5–6 fold reduction in blood vessel density [211]. This evidence suggests that tumor macrophages promote both angiogenesis and lymphangiogenesis that contribute, respectively, to hematogenous and lymphatic metastasis.

Another experimental approach that helped to examine the role of macrophages in lymphangiogenesis is systemic depletion of macrophages using clodronate liposomes (CDL). CDL depletion of CD11b^+^/LYVE-1^+^ TAMs in a model of ovarian cancer inhibited tumor-induced lymphangiogenesis by 50–75% (*p *< 0.05) [158]. Similarly, in an orthotopic model of bladder cancer, CDL depletion of VEGFR-3^+^ TAMs caused a statistically significant 74% reduction in LVD, and a similar decrease in lymphatic metastasis [212]. However, in contrast to other studies, elimination of TAMs affected only lymphatic vessels with no change in tumor blood vessel density [212]. In an orthotopic model of pancreatic cancer, CDL treatment resulted in the same extent of inhibition of LVD as anti-PlGF antibody discussed earlier [210], supporting the hypothesis that tumor lymphatic formation is primarily regulated by macrophages.

Not all studies, however, showed clear dependency between recruited TAMs and tumor lymphatics. For instance, tumor LVD was reduced by only 20% (*p* < 0.01) and LN metastasis was unchanged after CDL depletion of F4/80^+^ TAMs in a Rip1Tag2 insulinoma model [23]. The differences in study results might be due to the ability of CDL to effectively deplete all subsets of macrophages which seems to directly relate to their pro-lymphangiogenic effect. Currently, it is unclear whether a subset responsible for the pro-angiogenic effect overlaps with pro-lymphangiogenic sub-populations. Potentially, these effects can be mediated by distinct populations that might have differential sensitivity to CDL depletion, or be recruited by different chemokines. Alternatively, differential effects of TAMs depletion (or recruitment) on angio/lymphangiogenesis [23,212] could also relate to the variability in the composition of the tumor milieu in individual models [126,213]. In overall, despite some discrepancies in responses to macrophage depletion in various models, most studies provided supportive evidence for the contribution of TAMs to induction of tumor lymphangiogenesis.

### 3.4. Mechanisms of Macrophage-Mediated Contribution to Tumor Lymphangiogenesis

#### 3.4.1. Role of Pro-Lymphangiogenic Factors and Proteolytic Enzymes Produced by TAMs

Until recently, macrophage-dependent regulation of lymphangiogenesis was assumed to be performed mainly through secretion of paracrine mediators (Table 2). The main pro-lymphangiogenic mediators secreted by activated macrophages are VEGF-C, VEGF-D and VEGF-A. In addition, macrophages secrete a variety of pro-lymphangiogenic factors that contribute to this process indirectly by increasing the expression of VEGF-C, -D or -A. Several other products of activated macrophages such as PDGF [110], adrenomedullin [164], and HGF/SF [166] act directly on LEC that express corresponding receptors. The high affinity receptor for HGF, c-Met, is not only expressed on lymphatic endothelium but also elevated during inflammation [166]. Other factors (Table 2) are likely to enhance lymphangiogenesis through upregulation of VEGF-C or VEGF-D, although their pro-lymphangiogenic mechanisms may include both direct and indirect effects.

Activated macrophages also secrete high amounts of metalloproteinases and other proteases that facilitate degradation of extracellular matrix (ECM) and generation of active factors from matrix-embedded or soluble precursors. MMP-2, MMP-9, and heparanase exemplify some general facilitators of angiogenesis and lymphangiogenesis as both processes require remodeling of the surrounding cross-linked ECM in order to carve necessary space for new vessel formation. Plasmin, another product of inflamed macrophages typically present at high concentrations in solid tumors, is one of the proteases capable of proteolytical maturation of VEGF-C and VEGF-D propeptides [181]. The fully processed VEGF-C/-D can contribute to angiogenesis due to the acquired capacity to bind VEGFR-2 expressed on blood vessels [214]. Mature VEGF-C/-D factors also have increased affinity to VEGFR-3 which, together with the binding of VEGFR-2 expressed on LEC, may enhance the pro-lymphangiogenic response due to formation of VEGFR-2/VEGFR-3 heterodimers [82]. In summary, these findings demonstrate that macrophages activated by inflammation or tumor have very high potential to promote generation of new lymphatic vessels in a paracrine manner through secretion of direct and indirect pro-lymphangiogenic factors as well as proteases with lymphatic-growth promoting properties.

#### 3.4.2. Role of Macrophage-Derived LEC Progenitors (M-LECP)

The emerging evidence suggests that TAMs can promote lymphangiogenesis not only through paracrine mediators but also by differentiating into LEC progenitors that structurally contribute to the growing vasculature. Early LECP are BM-derived cells that express both myeloid progenitor and LEC markers, and are capable of undergoing lymphatic differentiation upon stimulation with an inflammatory stimulus. Evidence of differentiation includes the upregulation of LEC markers (Table 3) concomitant with downregulation of stem cell or progenitor markers, a process that leads to acquisition of the LEC phenotype marked by a physical contribution to the preexisting lymphatic vessels. Although LECP can be derived from several progenitor types, CD11b^+^ monocytes are identified as the main source in the majority of studies. Identification of macrophage-derived LECP (M-LECP) *in vivo* consists of visualization of double-stained cells that co-express myeloid markers (e.g., CD68, CD11b) and lymphatic-specific proteins such as LYVE-1, podoplanin, VEGFR-3, or Prox1 (Table 3).

**Table 3:** Reported expression of LEC markers by macrophage-derived lymphatic endothelial cell progenitors (M-LECP).

Gene name	Comments	Detection method	Ref.
VEGFR-3	Expressed by TG-stimulated peritoneal macrophages in culture	RT-PCR	[16]
	Detected in TG-induced peritoneal macrophages in culture	RT-qPCR	[19]
	Detected in bone marrow-derived macrophages in culture	RT-qPCR	[23]
	Expressed on culture CD11b^+^ bone marrow-derived cells that integrated into LV after reintroduction into mice	FACS, IHC	[53]
	Detected in activated peritoneal macrophages *in vivo* and in RAW264.7 macrophages *in vitro*	RT-qPCR, FACS	[30]
	Expressed by monocytes freshly purified from human blood	IHC, RT-PCR	[20]
Podoplanin	Detected on TG-stimulated peritoneal macrophages in culture	FACS, IHC	[16]
	Co-expressed with F4/80^+^ cells incorporated into LV *in vivo*	IHC	[19]
	Expressed by myeloid cells incorporated into LV *in vivo*	IHC	[23]
	Expressed on CD11b^+^ bone marrow-derived cells that integrated into LV *in vivo*	IHC, FACS	[53]
	Co-expressed on CD11b^+^ cells incorporated into LV *in vivo* and in activated peritoneal macrophages *in vivo*	IHC, RT-qPCR	[30]
	Expressed by cultured monocytes purified from human blood	IHC, RT-PCR	[20]
LYVE-1	Co-expressed on CD11b^+^ cells in LV *in vivo* and by TG-stimulated peritoneal macrophages in culture	IHC, FACS, IHC	[16]
	Co-expressed on F4/80^+^ cells incorporated into LV *in vivo*	IHC	[19]
	Co-expressed on F4/80^+^ cells incorporated into embryonic LS and LV	IHC	[52]
LYVE-1	Co-expressed on F4/80^+^ cells incorporated into LV *in vivo *	IHC	[23]
	Expressed on culture CD11b^+^ bone marrow-derived cells that integrated into LV after reintroduction into mice	FACS, IHC	[53]
	Co-expressed on CD11b^+^ cells incorporated into LV *in vivo* and inactivated peritoneal macrophages *in vivo*	IHC, RT-qPCR	[30]
	Expressed by monocytes freshly purified from human blood	IHC, RT-PCR	[20]
Prox-1	Co-expressed on CD11b^+^ cells in LV *in vivo *and by TG-stimulated peritoneal macrophages in culture	IHC, FACS, IHC	[16]
	Co-expressed with F4/80^+^ cells incorporated into embryonic LS and LV	IHC	[52]
	Expressed by myeloid cells incorporated into LV	IHC	[23]
	Expressed on cultured CD11b^+^ bone marrow-derived cells that integrated into LV after reintroduction into mice	FACS	[53]
	Co-expressed on CD11b^+^ cells incorporated into LV *in vivo*; Activated peritoneal macrophages *in vivo* expressed reduced levels compared to control group	IHC, RT-qPCR	[30]
Tie2	Activated peritoneal macrophages *in vivo* expressed reduced levels compared to control group	RT-qPCR	[30]

TG, thioglycolate; IHC, immunohistochemistry; LV, lymphatic vessels; FACS, fluorescence-activated cell sorting; LS, lymph sacs.

The clinical significance of circulating LEPC in cancer patients was first demonstrated by a subpopulation of CD133^+^/CD34^+^/VEGFR-3^+^ progenitors isolated from human fetal liver and cord blood [215]. In culture, these cells expressed a combination of endothelial and LEC-specific markers such as CD34, VE-cadherin, LYVE-1, and podoplanin [215]. The fraction of CD34^+^/VEGFR-3^+^ progenitors was low in healthy adults (~0.2%), but increased ~4 fold in lung cancer patients [215]. Moreover, the increase in this subpopulation correlated with LN metastasis (*p* < 0.01) and decreased overall patient survival (*p* < 0.01) [216]. Similar populations of LECP were also identified in experimental cancer and inflammatory models. For instance, when purified VEGFR-3^+^/CD34^+^ progenitors tagged by GFP were injected into recipient mice, they were recruited to the site of corneal injury and integrated into the inflamed lymphatic vessels [15]. A separate study found a substantial 15-fold increase in BM-derived podoplanin^+^ LECP in the circulation of tumor-bearing mice [53].

When isolated LECP were transferred into mice undergoing wound healing, they were recruited to ears and wounded skin of the recipient mice where they integrated into nascent lymphatic vessels [53]. Detection of incorporated LECP is challenging because of the low frequency of detectable structural contribution to the growing lymphatics (Table 4 and Table 5). For instance, adoptively transferred BM-cells comprised only 3–4% of LEC in the lymphatic vessels of Rip1Tag2 tumors [23]. Likewise, only 1–3% of lymphatic vessels in the liver, gastro-intestinal tissue, and kidney contained adoptively transferred GFP^+^ BM-derived cells that co-expressed VEGFR-3 and LYVE-1 [217].

Several inflammatory and tumor models also showed low frequency of adoptively transferred progenitors observed in only 5–8% of vessels (Table 4 and Table 5). The largest contribution reported thus far was from the model of LPS-induced peritonitis in which M-LECP at the peak of their recruitment were detected in ~50% of diaphragm lymphatic vessels [30]. This discrepancy in the number of adoptively transferred incorporated LECP might be related to the method of detection of GFP-labeled cells. Most studies utilized endogenous fluorescence of GFP protein that might fade upon fixation and other tissue handling procedures. Alternatively, GFP-positive cells can be identified by immunostaining with anti-GFP specific antibodies [30]. This technical approach highlights all GFP-positive cells thus eliminating the possibility of “missing” some cells due to denaturation or GFP inactivation during tissue handling. While these technical differences might explain some discrepancies in the reported degree of LECP incorporation, the occurrence of integration events raises little doubts as this has been extensively documented in models of both inflammation and cancer.

**Table 4:** LECP incorporation into inflamed lymphatic vessels.

Model	Cell origin or type	Tag	Markers	Time point of analysis	Integration of LECP into LV	Ref.
LPS induced peritonitis (m)	Native macrophages	none	CD11b, F4/80, LYVE-1	2 days ^a^	~50% of LV contained macrophages	[30]
LPS induced peritonitis (m)	RAW264.7 macrophages	GFP	CD11b, F4/80, LYVE-1, Podo	7 days ^a^	~20% of LV contained macrophages	[30]
Corneal micropocket (m)	CD34^+^/VEGFR-3^+^ BM-LECP	GFP	LYVE-1	1–4 days ^b^	~1.5% of lymphatic endothelium	[15]
Corneal micropocket (m)	CD34^+^/VEGFR-2^+^ BM-LECP	GFP	LYVE-1	1–4 days ^b^	~0.5% of lymphatic endothelium	[15]
Corneal micropocket (m)	Cultured Podo^+^ BM-MNC	DiI	LYVE-1	7 days ^b^	5.2% of LV contained DiI^+^ cells	[53]
Skin and ear wound (m)	Cultured Podo^+^ BM-MNC	DiI	LYVE-1	7 days ^b^	5.5% of LV contained DiI^+^ cells	[53]
Liver of irradiated mice ^c^	Hematopoietic stem cells	GFP	LYVE-1, VEGFR-3	1 month ^b^ & >1 year ^b^	2.4% & 3.2% of LV contained GFP^+^ cells	[217]
Gastro-intestinal tissue of irradiated mice	Hematopoietic stem cells	GFP	LYVE-1, VEGFR-3	>1 year ^b^	1.0–1.4% of LV contained GFP^+^ cells	[217]
Skin and ear wound (m)	Fresh Podo^+^ BM-MNC	DiI	LYVE-1	7 days ^b^	detected, not quantified	[53]
Corneal inflammation (m)	BM-MNC	GFP	CD11b, LYVE-1, Prox-1	3 or 7 days ^a^	detected, not quantified	[16]
Skin wound (m)	Native myeloid cells	none	F4/80, LYVE-1	5 days ^a^	detected, not quantified	[19]
Kidney transplant rejection (H)	Presumably BM	none	Y-chromosome, LYVE-1, Podo	N/A	4.5% of LEC were Y-chromosome^+^	[20]
Interstitial lung disease (H)	Native macrophages	none	CD68, Podo, VEGFR-3	N/A	~1.6 cells/mm of LV	[218]
Oncocerciasis (H)	Native macrophages	none	CD68, LYVE-1	N/A	detected, not quantified	[29]

LV, lymphatic vessels; (m), mouse; BM bone marrow; BM-MNC, bone marrow mononuclear cells; DiI (1,1'-dioctadecyl-3,3,3'3'-tetramethylindocarbocyanine perchlorate), dye used for cell tracking; Podo, podoplanin; (H), human; ^a^ time after onset of inflammation; ^b^ time after adoptive transfer of progenitor cells; ^c^ incorporation was also detected in non-irradiated animals.

**Table 5:** LECP incorporation into tumor-induced lymphatic vessels.

Model	Cell origin or type	Tag	Markers	Time point of analysis	Integration of LECP into LV	Ref.
Rip1Tag2 insulinoma (m)	BM Cells (-T cells)	GFP	Podo, LYVE-1, Prox1	5–7 weeks ^a^	3.5% GFP^+^/Prox1^+^^b^ 3.5% GFP^+^/LYVE-1^+^^b^ 3% GFP^+^/Podo^+^^b^	[23]
TRAMPC-1 prostate cancer (m)	BM cells (-T cells)	GFP	Podo, LYVE-1, Prox1	3–4 weeks ^c^	minimal GFP^+^/Prox1^+^ 2.8% GFP^+^/LYVE-1^+^^b^4.1% GFP^+^/Podo^+^^b^	[23]
B16-F1 melanoma (m)	Cultured Podo^+^ BM-MNC	DiI	LYVE-1	7 days ^a^	8.5% of LV contained DiI^+^ cells	[53]
T241 fibrosarcoma (m)	CD34^+^/VEGFR-3^+^ BM-LECP	GFP	LYVE-1	1–4 days ^a^	detected, not quantified	[15]
T241 fibrosarcoma (m)	CD34^+^/VEGFR-2^+^ BM-LECP	GFP	LYVE-1	1–4 days ^a^	detected, not quantified	[15]
Multiple intestinal neoplasia (m)	Hematopoietic stem cells	GFP	LYVE-1	6 weeks ^a^	detected, not quantified	[217]
Rip1Tag2 insulinoma (m)	BM Cells (-T cells)	GFP	LYVE-1, F4/80	5–7 weeks ^a^	detected, not quantified	[23]
Rip1Tag2 insulinoma (m)	CD11b^+^ cells	GFP	LYVE-1, Prox1	3 weeks ^a^	detected, not quantified	[23]
Rip1Tag2 insulinoma (m)	Common myeloid progenitor cells	GFP	Podo, LYVE-1	3 weeks ^a^	detected, not quantified	[23]
TRAMPC-1 prostate cancer (m)	Native CD11b^+^ cells	GFP	Podo, LYVE-1, Prox1	3–4 weeks ^c^	detected, not quantified	[23]
EL4 lymphoma & Lewis lung carcinoma (m)	Native myeloid cells	β-gal	CD31, Prox1	10–14 days ^c^	detected but lacked Prox1, not quantified	[219]

LV, lymphatic vessels; (m), mouse; BM, bone marrow; Podo, podoplanin; BM-MNC, bone marrow mononuclear cells; DiI (1,1'-dioctadecyl-3,3,3'3'-tetramethylindocarbocyanine perchlorate), dye used for cell tracking; (H), human; ^a^ time after adoptive transfer of progenitor cells; ^b^ percentage of LEC; ^c^ time after tumor initiation.

##### 3.4.2.1. Incorporation of M-LECP into Inflammation-Induced and tumor Lymphatic Vessels

The main evidence supporting M-LECP lymphatic vascular integration is derived from mouse inflammatory models including those induced by LPS [30], radiation [217], wounding [19], or corneal surgery [15] (Table 4). In most studies, LECP incorporation has been shown by tracking BM-derived progenitors using GFP, β-gal, or a fluorescent dye marker, DiI (Table 4). The ability to trace implanted BM-derived cells in conjunction with double or triple staining using antibodies against myeloid and lymphatic-specific proteins enables identification and quantification of LECP incorporation into lymphatic vasculature. For instance, CD11b^+^ macrophages [16,30] and/or F4/80^+^ [19,30] cells were detected within lymphatic structures that co-expressed the lymphatic markers LYVE-1 [16,19,30] or Prox1 [16]. The percent of incorporation varies between 1–5%, although the recently published peritonitis model reported LECP integration as high as 50% [30]. Some studies detected incorporated tagged cells but did not quantify the rate of incorporation [19,53]. Interestingly, in the model of inflamed cornea performed in mice expressing LacZ under the promoter of Tie2, activated lymphatic endothelium was not stained by β-gal [16]. This suggested that Tie2-expressing macrophages, albeit playing a major role in angiogenesis [220], might not be the major contributors to lymphangiogenesis.

A cell autonomous contribution of M-LECP to lymphatic endothelium was also demonstrated in inflammatory human pathologies such as oncocerciasis [29], and interstitial lung disease [218] (Table 4). Nodules that develop around the parasitic filarial nematode *Onchocerca volvulus *share characteristics with tumors such as a mixed Th1/Th2 inflammatory response, predominately monocytic infiltrate, abundance of angio/lymphangiogenic factors, and growth of blood and lymphatic vessels [29]. Indeed, within these nodules, CD68^+^/LYVE-1^+^ macrophages co-localized with the endothelial layer of newly-formed lymphatics [29]. In case of the human interstitial lung disease, CD68^+^/D2-40^+^ or CD14^+^/D2-40^+^ macrophages co-localized with the lymphatic endothelial layer of newly formed vessels in intra-alveolar fibrotic lesions at a rate of ~1.6 cells per millimeter of endothelium [218].

Finally, a notably elegant study examining lymphangiogenesis in gender-mismatched renal transplant rejection identified Y-chromosome and Prox1-positive LEC progenitors in 4.5% of lymphatic vessels in female recipients [20]. Cumulatively, this evidence strongly supports an active pro-lymphangiogenic role of circulating BM-derived myeloid progenitors in inflammatory lymphangiogenesis in human adults.

Studies on M-LECP’s role in tumor-induced lymphangiogenesis are lagging behind examination of their role in the inflammatory field. Nevertheless, incorporation of M-LECP into tumor-induced lymphatic vessels has been detected in seven cancer mouse models (Table 5). For instance, TAMs that were positive for CD11b^+^ and F4/80^+^ as well as for LYVE-1 and stabilin-1 [221] were found in B16-F1 melanoma and Rip1Tag2 insulinoma models [28]. In B16-F1 melanoma tumors, LYVE-1 and F4/80 co-localized with lymphatic structures, suggesting that a subset of TAMs became part of the tumor lymphatic endothelium [28]. In another study of B16-F1 tumors, BM-derived podoplanin^+^/CD11b^+^ mononuclear cells activated *in vitro *were incorporated into 8.5% of LYVE-1^+^ lymphatic vessels after adoptive transfer [53]. In the model of Rip1/Tag2 insulinoma, adoptively transferred GFP-tagged BM-derived LECP were detected in 3–4% of Prox1^+^, LYVE-1^+^, or podoplanin^+^ peritumoral lymphatic vessels [23]. Lymphatic vascular integration of BM-derived myeloid cells was confirmed by triple-staining for GFP, LYVE-1 and F4/80, and by lineage tracing experiments that demonstrated integration of FACS-sorted GFP^+^/CD11b^+^ cells and common myeloid progenitors [23].

As illustrated by Gordon *et al. *[219], the field is still divided with regard to identification of macrophages detected in the wall of the nascent lymphatics. Some believe that those are macrophage-derived LECP expressing markers of both lineages while others view these cells as macrophages transmigrating through the lymphatic vessel wall. Indeed, diapedesis cannot be ruled out as there are not yet images from high resolution confocal, electron, or intravital microscopy that show macrophages resting in the lymphatic endothelium and expressing morphological characteristics of LECs. However, several lines of evidence strongly advocate for structural contribution of myeloid cells to the growing lymphatics: (1) As described in detail in the next section, multiple studies reported transcriptional reprogramming (transdifferentiation) of a subset of macrophages destined to become LECP. These cells synthesize, *de novo*, quintessential markers of lymphatics such as: VEGFR-3, LYVE-1, podoplanin, (Table 3) and nearly 30 other LEC proteins [30]. No evidence supports the concept that traversing the lymphatic barrier necessitates acquisition of LEC-specific markers whereas it is widely accepted that lineage transdifferentiation mandates genetic reprogramming appropriate to the new phenotype. Therefore, novel expression of LEC-specific proteins in macrophages is much more likely to indicate a switch to the lymphatic lineage than acquisition of a LEC phenotype during transmigration through lymphatic vessels; (2) M-LECP incorporated within the lymphatic vessel wall co-express myeloid/LEC markers. Reported images from Z-stack analysis using confocal microscopy [16,53] show M-LECP with dual lineage markers forming multicellular structures in the same plane as the lymphatic endothelial layer. These images are much more consistent with the concept of integrated M-LECP within the lymphatic wall rather than snapshots of singly transmigrating myeloid cells [222] that show discrete planar separation between lymphatic and myeloid markers; (3) In contrast to dendritic cells, macrophages are infrequently found in the lymph under quiescent conditions [223]. Moreover, macrophage egress from inflamed tissues through lymphatic vessels coincides with resolution of inflammation [224]. In contrast, drastic increase of M-LECP incorporation into lymphatics is detected at the early stages of inflammation, immediately after their recruitment to the site through blood vessels [16,19,30,53]. Thus, the peaks of M-LECP integration and possible macrophage diapedesis through lymphatics are temporally distinct.

##### 3.4.2.2. Transdifferentiation of Macrophages into M-LECP

Transdifferentiation is the reprogramming of a fully differentiated cell that induces development of traits and functions typically found in cells from another lineage [225]. Several studies indicate that activation of macrophages by inflammatory stimuli triggers their transdifferentiation into M-LECP as evidenced by increased expression of lymphatic genes and downregulation of myeloid markers. For example, activated macrophages express quintessential lymphatic markers such as: VEGFR-3, LYVE-1, Podoplanin, and variably Prox-1 (see Table 3). A recent, particular in depth study demonstrated that treatment of mice with LPS increased VEGFR-3 expression in several subsets of CD11b^+^ monocytes [30]. To comprehensively characterize the CD11b^+^/VEGFR-3^+^ monocytes, their expression of 54 genes typically expressed by LECs was evaluated by RT-qPCR [30]. Compared with the CD11b^+^/VEGFR-3^−^ population, 29 genes were upregulated including lymphatic-specific markers including NRP-2, podoplanin, Sox17, VEGF-C and VEGFR-3. Notably, the major lymphatic marker LYVE-1 increased 41-fold. The ability of inflamed macrophages to express lymphatic-specific markers was also shown *in vitro* using mouse macrophage line RAW264.7. LPS-treated RAW264.7 cells exhibited a ~10 fold increase in VEGFR-3 mRNA expression followed by a 32-fold upregulation of surface protein [30]. These findings support the notion that some subsets of macrophages have sufficient plasticity to acquire the lymphatic phenotype [30].

The loss of myeloid markers has also been observed *in vivo *following the incorporation of M-LECP into lymphatic structures [19,30]. For example, co-localization of myeloid markers in the lymphatic vessels of diaphragm peaked at day 2 (~50%) and returned to basal levels by day 5 after LPS-induced peritonitis [30]. This is significant because the brevity of the time window, when M-LECP display double identity markers, argues for the necessity to perform detailed kinetic studies to quantitatively assess contribution of M-LECP to growing vasculature. The narrow window and rapid loss of myeloid markers might also explain some discrepancies in the studies that analyzed M-LECP in different time points after onset of inflammation or tumor implantation (Table 4 and Table 5, see Time point of analysis). This point should also be considered for analysis of clinical studies, because in contrast to experimental models, the onset of inflammatory and malignant processes in human subjects is largely unknown.

Reprogramming of activated macrophages into LEC-like cells is further supported by matrigel-promoted tube formation assays. Matrigel is derived from the Engelbreth Holm-Swarm sarcoma and contains a complex mixture of ECM proteins, growth factors, and cytokines that mimics the *in vivo *extracellular environment [226]. The mixture is commonly used to analyze *in vitro* activation and differentiation of EC [226] and EPC [227,228,229], typically determined by the ability of cells to form tube-like structures. This approach has been used to demonstrate endothelial-like morphological changes in macrophages accompanied by altered molecular profiles representing the lymphatic phenotype. For instance, immunofluorescent analysis of thioglycolate-activated peritoneal macrophages revealed the co-expression of CD11b and lymphatic markers Prox1, podoplanin, and LYVE-1 [16]. When seeded on matrigel, these magcrophages formed tube-like structures positive for LYVE-1 and podoplanin [16]. Similarly, activated CD45^+^/CD14^+^/CD11b^+^ macrophages, isolated from bronchoalveolar lavage fluid of interstitial lung disease patients, formed LYVE-1^+^/podoplanin^+^ vessel-like structures [230]. In contrast, macrophages from healthy patients expressed low levels of LYVE-1 and did not form tubular structures or express podoplanin [230]. Likewise, murine BM-derived CD11b^+^/F4/80^+^ macrophages activated by LPS formed podoplanin^+^ tube-like structures whereas untreated macrophages neither formed tubes nor expressed podoplanin [23]. Furthermore, gene profile comparative analysis of macrophages that formed or did not form tubes revealed substantial differences in expression of lymphatic and myeloid genes. Cells able to form tubes showed marked upregulation of lymphatic genes such as LYVE-1, Prox1, VEGFR-3, FoxC2, and FGFR1/2, along with downregulation of monocyte/hematopoietic markers CD45 and CX3CR1. This finding is reminiscent of similar pro-lymphatic changes in gene expression in LPS-treated RAW264.7 macrophages *in vitro* and endogenous myeloid cells in LPS-treated mice [30].

##### 3.4.2.3. Evidence of Lymphvasculogenesis Induced by Adult M-LECP

While little doubt exists that lymphvasculogenesis contributes to the formation of embryonic lymphatic system [49,50,52], the role of this process in adults is a subject of debate. The most convincing evidence supporting postnatal lymphvasculogenesis came from a model of corneal inflammation [16]. This model is well-suited for analyzing lymphvasculogenesis because the normal cornea is avascular as blood and lymphatic vessels neatly terminate in the peripheral limbus. Additionally, the cornea responds to inflammation by generating new blood and lymphatic vessels that can be clearly visualized in the thin, transparent tissue. Lymphvasculogenesis was demonstrated in cultured explants from central cornea that lacked pre-existing lymphatic vessels, but nonetheless, had the capacity to develop LYVE-1^+^ structures after stimulation with an inflammatory mediator, IL-1β [16]. In animals, surgically induced corneal inflammation prompted recruitment of dual-positive CD11b^+^/LYVE-1^+^ and CD11b^+^/Prox1^+^cells that subsequently incorporated into newly-formed lymphatic structures. Importantly, these structures often lacked connections with the pre-existing limbal vasculature, suggesting that they were formed *de novo *[16]. Additional evidence supporting the vasculogenic potential of M-LECP was demonstrated in a model of LPS-induced peritonitis [30]. LPS-activated RAW264.7 macrophages were first investigated *in vitro* where they showed clear evidence of reprogramming into LEC-like cells [30]. Next, activated GFP-tagged RAW264.7 macrophages were injected into either control or LPS-treated mice. After seven days, the lymphatic vessels of the diaphragm were examined for signs of RAW264.7 incorporation. Whereas control mice showed no recruitment of GFP-positive cells, diaphragms of LPS-treated mice displayed massive clusters of GFP^+^/CD11b^+^/LYVE-1^+^ cells that intimately interacted with inflamed peritoneal lymphatic vessels [30]. Moreover, vessel-like structures were observed within the RAW264.7 macrophage clusters that were distinctly, spatially separated from the diaphragmal vessels, suggesting *de novo* formation of these structures [30]. Taken together this evidence indicates that lymphvasculogenesis can occur postnatally, and that macrophages play an important cell autonomous role in this process.

The prerequisite for macrophage ability to form new lymphatic vessels is the acquisition of LEC phenotype signified by *de novo* expression of lymphatic-specific genes (Table 3). This may occur through transdifferentiation described above, or through the related differentiation process in which stem or progenitor cells with a relatively high developmental potential acquire new traits according to commitment of their lineages. Whether macrophages differentiate or transdifferentiate into LEC is still an open question as the existing evidence might be interpreted as supportive for both mechanisms. It is also possible that different subtypes of macrophages and monocyte progenitors may undergo either reprogramming process, and yet yield LECP with similar genetic make-up and functional properties. Future studies employing lineage tracing of BM-derived myeloid progenitors and novel transdifferentiation model systems [30] might resolve this fundamental question in the lymphatic biology.

## 4. Other BM-Derived Progenitors that Might Contribute to Tumor and Inflammatory Lymphangiogenesis

Although myeloid cells are the most frequently reported as sources of LECP, other types of progenitors have also been implicated in this process. These include CD34-positive hematopoietic stem cells (HSC) [215,217], mesenchymal stem cells (MSCs) [231], and adipose-derived stem cells (ASC) [232] (Table 4 and Table 5). Adoptively transferred GFP^+^ c-kit^+^/sca-1^+^/Lin^−^ [217] and GFP^+^/CD34^+^ [15] HSC that expressed lymphatic markers were found integrated into the lymphatic endothelium in several models of inflammation [15,217] and tumor induced lymphangiogenesis [15,217]. MSCs were shown to upregulate VEGFR-3, Prox1, and podoplanin in response to VEGF-C and increase the re-growth of severed lymphatic vessels when implanted into wounded mouse tails [231]. VEGF-C also induced transition toward the lymphatic phenotype in ASC by increasing LYVE-1 and Prox1 expression while reducing transcripts of the stem markers Sca-1 and CD29 [232]. Furthermore, matrigel implanted and VEGF-C treated ASC placed *in vivo* formed podoplanin^+^ vessel-like structures indicating their structural potential to contribute to growing vasculature [232]. It should be noted that the majority of studies examined expression of markers in the newly-formed structures, but not the functional capacity of the vessels. However, collectively, these studies suggest that several populations of stem and progenitors might contribute to growing lymphatics, particularly in the presence of a high local concentration of VEGF-C that appears to be the main driving force for acquisition of the lymphatic phenotype in VEGFR-3^+^ cells.

## 5. Conclusions

**Figure 1:**
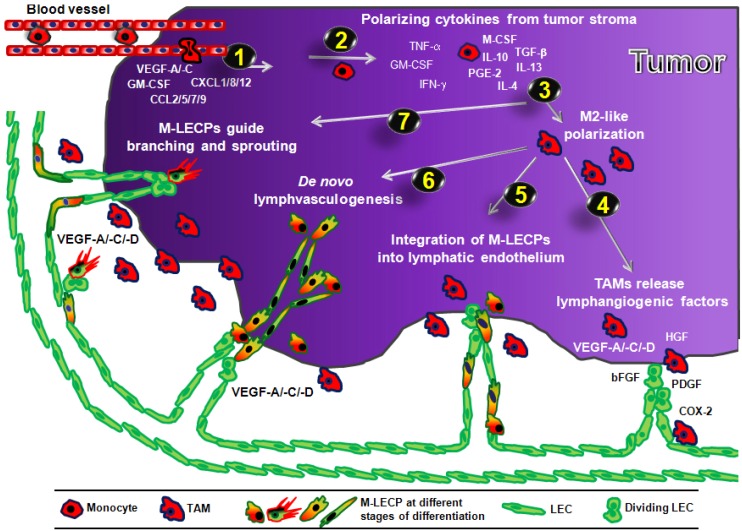
Potential roles of TAMs and macrophage-derived LECP in tumor lymphangiogenesis. Schematic representation of known and potential roles of tumor-associated macrophages that can promote lymphangiogenesis. (1) Tumors produce many chemotactic agents such as CCL2, GM-CSF, CXCL1, and VEGF-A that recruit macrophages from circulation or adjacent tissues; (2) Monocytes mature into macrophages in response to activating cytokines secreted by tumors. These include cytokines associated with the M1/Th1 immune response such as TNF-α, GM-CSF, and INF-γ; and those related to the M2/Th2 immune response including IL-10, TGF-β, and M-CSF; (3) Tumor cytokine milieu polarizes macrophages towards the pro-tumorigenic M2 phenotype (TAMs) that has been correlated with LN metastasis in humans (Table 2); (4) TAMs stimulate lymphangiogenesis in a paracrine manner by producing multiple lymphangiogenic factors that stimulate proliferation and migration of LEC (Table 2); (5) TAMs have been shown to differentiate into M-LECP (Table 3) and structurally contribute to the tumor lymphatic endothelium (Table 5); (6) In inflammatory models, M-LECP have been shown to coalesce and form *de novo* lymphatic vessels suggesting that a similar process might occur in tumors; (7) M-LECP have been also shown to incorporate into chord structures at branch points and at the tips of growing sprouts *in vitro* suggesting that they guide branching and sprouting [23]. By a similar mechanism, M-LECP might guide the growing tips of lymphatic sprouts while migrating towards a chemo-attractant produced by the tumor.

Macrophages have long been implicated as the major regulators of lymphangiogenesis primarily through secretion of paracrine mediators such as VEGF-C, VEGF-A and VEGF-D. The new evidence suggests that macrophages can also promote lymphangiogenesis by other mechanisms including transdifferentiation into LECP that structurally contribute to and provide branching directions for newly-constructed lymphatic vessels (Figure 1).

Although an impressive body of evidence supports the emerging concept of M-LECP and their significance in inflammatory and tumor lymphangiogenesis, this new field of study can benefit from additional approaches including *in vivo* live imaging and lineage tracing experiments. Nevertheless, lymphatic vessel-embedded cells with double myeloid/lymphatic identity are frequently detected specifically at inflammatory sites strongly arguing for the central role of macrophages in LECP generation and their important functions in promoting lymphatic growth. However, the mechanisms of LECP recruitment to inflammatory sites, interactions with lymphatic endothelium prior to vascular integration, and importantly, post-integration functions are currently unknown. Elucidation of these questions not only will advance understanding of postnatal lymphangiogenesis but will also shed light on fundamental mechanisms of lineage plasticity allowing formation of new structures in adults. Better understanding of cell-autonomous pro-lymphangiogenic roles of inflamed macrophages may suggest new therapeutic approaches for correcting lymphatic dysfunction in human disorders.
